# Studying Implicit Attitudes Towards Smoking: Event-Related Potentials in the Go/NoGo Association Task

**DOI:** 10.3389/fnhum.2021.634994

**Published:** 2021-02-05

**Authors:** Tobias A. Wagner-Altendorf, Arie H. van der Lugt, Jane F. Banfield, Jacqueline Deibel, Anna Cirkel, Marcus Heldmann, Thomas F. Münte

**Affiliations:** ^1^Department of Neurology, University of Lübeck, Lübeck, Germany; ^2^Department of Cognitive Neuroscience, Faculty of Psychology and Neuroscience, Maastricht University, Maastricht, Netherlands; ^3^Department of Neuropsychology, Institute of Psychology, Faculty of Natural Sciences, Otto von Guericke University, Magdeburg, Germany; ^4^Institute of Psychology II, University of Lübeck, Lübeck, Germany

**Keywords:** Go/NoGo association task (GNAT), N200, EEG, ERP, implicit association, attitudes, cigarette smoking, addiction

## Abstract

Cigarette smoking and other addictive behaviors are among the main preventable risk factors for several severe and potentially fatal diseases. It has been argued that addictive behavior is controlled by an automatic-implicit cognitive system and by a reflective-explicit cognitive system, that operate in parallel to jointly drive human behavior. The present study addresses the formation of implicit attitudes towards smoking in both smokers and non-smokers, using a Go/NoGo association task (GNAT), and behavioral and electroencephalographic (EEG) measures. The GNAT assesses, *via* quantifying participants’ reaction times, the strength of association between a target category and either pole of an evaluative dimension (positive or negative). EEG analysis is performed to determine the temporal course of the event-related potential (ERP) components underlying Go/NoGo decisions and implicit attitude formation. Both smokers and non-smokers showed prolonged reaction times to smoking-related pictures when the pictures were coupled with positive evaluative words (“incongruent condition”). This indicates negative implicit attitudes towards smoking in both groups alike at the time point of the behavioral response (600–700 ms post-stimulus). However, only the non-smokers, not the smokers, were found to show a delay of the N200 component in the incongruent condition. This is interpreted as reflecting ambivalent or even positive implicit attitudes towards smoking in the smoker group at the time point of the N200 (300–400 ms post-stimulus). Our study thus provides evidence for the hypothesis that implicit attitudes are subject to changes within several hundred milliseconds after stimulus presentation, and can be altered in the course of their formation.

## Introduction

Smoking is the main preventable risk factor for several important causes of death. Yet even though the negative consequences of addictive behaviors such as cigarette smoking are well documented, and subject to mass media campaigns and legislation enforcing, e.g., the use of warning labels, about one in four adults in the European Union continues to smoke (Eurobarometer, 2017; Pesce et al., 2019).

It has been argued that two different cognitive systems control addictive behaviors: an automatic (i.e., implicit) system and a controlled (i.e., reflective) system (e.g., Wiers et al., 2007; Evans, 2008). The automatic system is thought to play an important role in the development and maintenance of addiction (Robinson and Berridge, 2003; Wiers et al., 2010). However, more recently, it has been pointed out that there is no strict separation between automatic and controlled systems, and that a more nuanced view of the role that automatic processes play in drug-related behaviors is needed, as an automatic system may include not only smoking-related associations but also complex smoking-related beliefs (Gladwin et al., 2011; Tibboel et al., 2017).

Studies investigating the automatic or implicit associations of participants addicted to cigarettes or alcohol have highlighted a paradox: while smokers and drinkers may continue to engage in addictive behaviors and express positive explicit beliefs about smoking or drinking, they may also (just like non-smokers and non-drinkers) show implicit negative attitudes towards these behaviors (Swanson et al., 2001; Wiers et al., 2002; Sherman et al., 2003; Houben et al., 2006; Wiers and De Jong, 2006; Glock et al., 2014; Cui et al., 2019). Studies investigating the shift of attention to smoking-related stimuli have revealed that smokers, independently of craving, were actively avoiding the smoking-related images (Donohue et al., 2016).

Given this clear evidence for negative implicit attitudes towards smoking in non-smokers and smokers alike, other studies, however, have found smokers’ attitudes towards smoking to be less negative than non-smokers’ attitudes (but still negative; Huijding et al., 2005; Perugini, 2005), to be ambivalent (Robinson et al., 2005), or even positive (Ren et al., 2019). Lee et al. (2017) report that smokers who developed a more negative attitude towards smoking were more likely to quit smoking, even controlling for explicit motivation to quit.

Perugini (2005) found that only the explicit attitude measure significantly predicted whether someone is a smoker or a non-smoker, arguing however that implicit and explicit attitudes closely interact in influencing behavior. Building on the reflective-impulsive model of social behavior proposed by Strack and Deutsch (2004), it is assumed that the automatic-implicit and the reflective-explicit systems operate in parallel, mutually influencing each other, and jointly guiding human behavior.

A behavioral method that is used to study subjects’ implicit attitudes or associations is the Go/NoGo association task (GNAT; Nosek and Banaji, 2001). It can be used to measure the strength of association between a target category and either pole of an evaluative dimension (i.e., positive or negative). In the GNAT, participants are required to map the same response (Go or NoGo) to stimuli that belong to a target category (as opposed to a comparison category) and words representing one pole of an evaluative dimension. The pairing can be evaluatively congruent (e.g., make a response to a “good” category picture or a positive word) or incongruent (e.g., make a response to a “bad” category picture or a positive word). It has been consistently found that response latencies are driven by the (in)congruency, i.e., participants respond more quickly to target pictures when the instructions pair good target pictures with positive evaluative words or bad target pictures with negative evaluative words (the congruent conditions). The idea is that it is easier to map a category and an evaluation onto the same response (i.e., to respond or to withhold the response, respectively) if they are congruent, that is, previously linked by implicit attitudes.

In the present study, groups of 15 smokers and 15 non-smokers performed a GNAT. Pictures related to smoking (e.g., of a cigarette pack or an ashtray) and neutral/non-smoking pictures (e.g., of pencils or a hole puncher), as well as positive or negative words (e.g., “flower” and “holiday,” or “bomb” and “threat,” respectively), were presented consecutively in a randomized order. Participants had to either respond *via* a mouse click (“Go”) or withhold their response (“NoGo”), depending on the instructions. The study aimed to examine the implicit attitudes of smokers and non-smokers towards smoking, as reflected both in the behavioral data and in electroencephalography (EEG) potentials during the GNAT.

When using the GNAT in an event-related potential (ERP) task, NoGo stimuli give rise to the “NoGo N200” ERP component—a negative potential at ca. 200–400 ms after the stimulus and maximal over frontal regions of the scalp (see, e.g., Jodo and Kayama, 1992 and Nieuwenhuis et al., 2003, for Go/NoGo tasks; see, e.g., Banfield et al., 2006 and Wu et al., 2014, for GNAT experiments). It has been suggested that the N200 during GNAT reflects response conflict monitoring by the ACC (Nieuwenhuis et al., 2003), i.e., conflict between the prepotent (Go) response and the required withholding of the response. Gajewski and Falkenstein (2013) have reported, in a Go/NoGo task, that the latency but not the amplitude of the NoGo N200 is susceptible to task complexity, and infer that the NoGo N200 either reflects the inhibition of a premature response plan or the detection or resolution of response conflict between Go and NoGo response tendencies.

Importantly, the temporal course of the NoGo N200 component is dependent on the congruency of the target category and the evaluative dimension. In runs that required the same response for congruent stimulus pairings, the NoGo N200 appeared earlier than in runs with incongruent stimulus pairings. This was found in GNAT studies where participants had to characterize fruit and bug words (Banfield et al., 2006), pictures of young and elderly people (van der Lugt et al., 2012), or words concerning oneself and others (Wu et al., 2014).

In the present study, we expected—as shown in many preceding studies—to find clear signs for negative implicit attitudes towards smoking in non-smokers—that is, behaviorally, prolonged reaction times and higher error rates in the incongruent compared to the congruent condition; and, electrophysiologically, a NoGo N200 ERP component which is delayed in the incongruent compared to the congruent condition. For smokers, there is conflicting evidence so far in terms of negative, neutral, or positive attitudes towards smoking. It has to be noted, however, that EEG and behavioral measures address different time windows of (implicit) attitude formation (Banfield et al., 2006; van der Lugt et al., 2012); and we thus expected that the event-related EEG potential would give us information on earlier automatic associations than the behavioral implicit-association test. If the formation of implicit attitudes is subject to early changes, one thus might expect differing indications of implicit attitudes in EEG and behavioral measures.

## Materials and Methods

### Participants

A total of 30 healthy, right-handed participants (16 females, 14 males), aged 19–30 years (mean: 23.6 years) took part in the study. All participants were German native speakers. Fifteen participants were smokers, smoking 7–25 cigarettes per day (mean: 14.3) for at least 3 years (mean: 7.7). They did not smoke within 2 h before the start of the experiment. Fifteen participants were non-smokers who had never smoked. Before taking part in this study, participants signed an informed consent form. The study was approved by the local ethics committee. Participants were paid 6.50 € per hour for participation.

### Procedure

Positive and negative German words (40 in each category; see Supplementary Appendix A for a full list) were matched for length and word frequency (Baayen et al., 1993) and were chosen for unambiguous valence. A comparison of the word stimuli in both conditions using the scientific database SUBTLEX-DE (Brysbaert et al., 2011) showed no difference in lexical frequency of the stimuli as indicated by lgSUBTLEX value (mean positive: 2.25; mean control: 2.19; *p* = 0.74, unpaired *t*-test). Word stimuli were presented in black (Arial, 16 pt) against a white background. Colored pictures related to smoking or of neutral/non-smoking content (40 in each category; see Supplementary Appendix B for examples) were used. Smoking-related pictures showed objects such as cigarettes, ashtrays, and lighters. Neutral pictures showed everyday objects such as kitchen utensils, tools, and pencils. Neutral pictures were matched for shape and color to smoking pictures. All pictures were cropped to the same specification (200 × 200 pixels) and presented against a white background. Subjects were seated 90 cm from the video-screen.

Participants performed eight experimental blocks (two blocks per condition, four conditions; see below). Each block contained 176 trials, comprising the abovementioned 160 experimental stimuli preceded by an additional 16 practice trials. The same word and picture stimuli were arranged in random order within each block. At the start of each trial, a fixation cross was centrally presented for a random duration between 500 and 1,000 ms, immediately followed by either a word or a picture which remained on the screen for 1,500 ms, followed again by a fixation cross for 500–1,000 ms until the end of the trial. Participants were required to make a Go/NoGo decision on the presentation of each stimulus, depending on the instructions given at the start of the block, and again as a reminder at the end of the practice trials.

Four experimental conditions determined the decision: two congruent conditions (“press if a smoking-related picture or negative word”/“press if a neutral picture or positive word”) and two incongruent conditions (“press if a smoking-related picture or positive word”/“press if a neutral picture or negative word”). The order of blocks was rotated and counterbalanced across participants.

Participants were instructed to respond as quickly as possible, according to the given instruction, *via* a mouse button press (“Go”), or by withholding their response (“NoGo”), respectively. The button was pressed with the index finger, for four blocks with the right hand and another four blocks with the left hand. For half of the participants this order was reversed (i.e., starting with the left hand).

**Figure 1:**
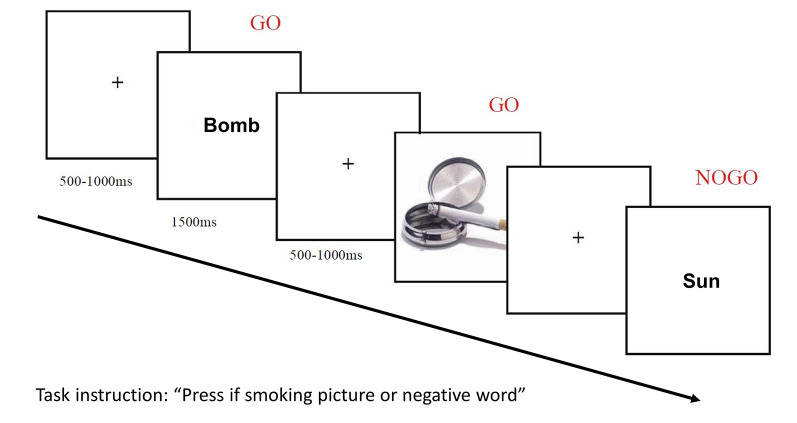
Schematic drawing of the trial procedure of the Go/Nogo association task (GNAT). The participant views a random series of stimuli comprising pictures (smoking-related and neutral) and words (positive and negative) with the task of pressing a button as fast as possible for one picture category (in this example smoking-related) and one word category (in this example negative words), while withholding responses for the other categories.

See Figure 1 for a schematic drawing of the trial procedure.

### Data Acquisition

The EEG was recorded from 29 scalp electrodes against a reference at the left mastoid process. The electrophysiological signals were recorded with a bandpass of 0.01–70 Hz (half-amplitude cutoffs) and digitized at a rate of 250 Hz. Electrode impedances were kept under 6 KΩ. Vertical (vEOG) and horizontal (hEOG) electrooculograms were monitored from electrodes placed below the right eye, and at the left and right outer canthi, respectively. EEG and EOG data were recorded with Acquire^®^ software. For EEG analysis, EEGLAB (Delorme and Makeig, 2004) and ERPLAB (Lopez-Calderon and Luck, 2014) were used. Stimulus locked data were calculated with an epoch length of 1,300 ms (−300 ms before to 1,000 ms after word/picture presentation). The defining conditions for the stimulus onset were the correct trials for congruent/incongruent Go and NoGo conditions, separated for picture and word stimuli. To remove ocular artifacts, independent component analysis (ICA) was used. To account for non-ocular artifacts such as amplifier blocking or sudden jumps in amplitude the “moving window peak-to-peak threshold” function was used, with a threshold potential individually adjusted for each participant after visual inspection of long stretches of EEG. Epochs containing these artifacts were excluded from the analysis. On the mean, 3.0% of trials were discarded due to the high artifact rate. ERPs were filtered with a 40 Hz low-pass filter. For baseline correction, the baseline was defined as the interval from −100 to 0 ms. From the resulting data, averages for each segment and participant were determined, and subsequently, grand averages were calculated for all participants. Behavioral data, i.e., reaction times and error rates, was recorded with Presentation^®^ software.

### Statistical Analysis of Behavioral Data

Participants’ reaction times and error rates to smoking-related pictures and neutral/non-smoking pictures were analyzed with open-source tool Jamovi^®^ (version 1.0.7^1^) and GraphPad Prism (version 6, Graphpad Software Inc., La Jolla, CA, USA), using repeated-measures ANOVA with Huynh–Feldt correction and, as indicated, paired *t*-tests. Uncorrected *F*, but corrected *p*-values are reported. Reaction times above 2,000 ms—which occurred very rarely (<0.1% of trials)—were not considered for analysis (cut-off).

### Statistical Analysis of ERP Data

Participants’ ERPs following the presentation of the smoking-related pictures were analyzed.

Visual inspection of the grand average ERP waveforms of all participants revealed frontocentral negativity beginning at ca. 250 ms that was more pronounced in the NoGo condition (the NoGo N200 component). This was quantified using repeated-measures ANOVA to test for differences between the four conditions in the mean amplitude between 250 and 400 ms. Because the NoGo N200 is known to have a frontal maximum, we restricted the analysis to electrodes F3/F4/Fz.

Visual inspection of the grand average ERP waveforms of non-smokers and smokers depicted separately revealed that the frontocentral NoGo N200 effect was clearly delayed in the incongruent condition in the non-smoker group, but only slightly in the smoker group. This was quantified by analyzing the NoGo incongruent-minus-congruent difference waveforms for both groups using repeated measures ANOVA and unpaired *t*-tests.

The level of significance was set at *p* < 0.05 (*), *p* < 0.01 (**), *p* < 0.001 (***) and *p* < 0.0001 (****).

## Results

### Behavioral Data

Analysis of the mean reaction times to smoking-related pictures revealed that both groups showed faster reaction times in the congruent compared to the incongruent condition; smokers showed slightly slower reaction times (non-smoker group: 564 vs. 624 ms; smoker group: 597 vs. 657 ms). Repeated measures ANOVA for reaction times revealed a highly significant main effect of congruency (*F*_(1,14)_ = 41.72; *p* < 0.001), while there was neither a significant main effect of group (*F*_(1,14)_ = 2.02; *p* = 0.18) nor a congruency by group interaction (*F*_(1,14)_ = 0.001; *p* = 0.97). A paired *t*-test revealed highly significant differences between congruent and incongruent conditions in both groups (*p* < 0.001).

**Figure 2:**
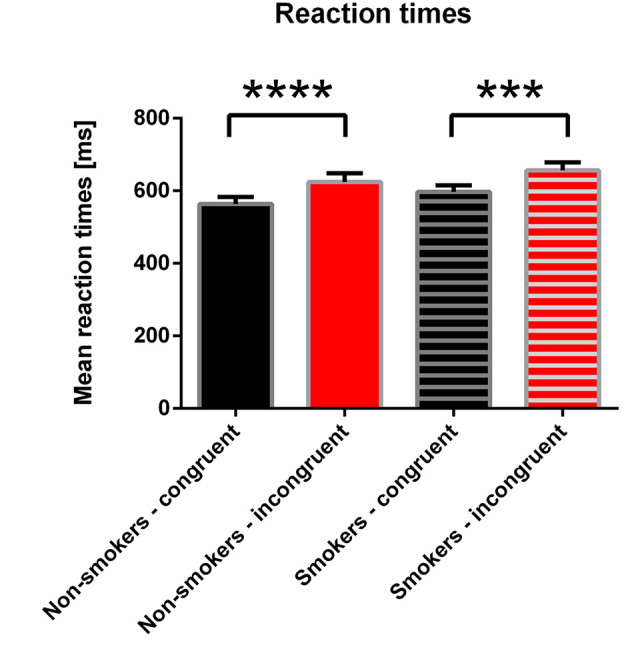
Reaction times for non-smokers and smokers in classifying smoking-related pictures (Go condition). Note that both groups show slower reaction times in the incongruent condition. The difference within the groups is highly significant (*p* < 0.0001 and *p* = 0.0004, for non-smokers and smokers, respectively; paired *t*-test). *** *p* < 0.001, **** *p* < 0.0001.

See Figure 2 for reaction times to smoking-related pictures.

Error rates, in general, were low; being lower in the congruent compared to the incongruent condition for both groups (non-smokers: 0.92% vs. 1.34%; smokers: 1.17% vs. 1.75%). rmANOVA revealed a significant main effect of congruency (*F*_(1,14)_ = 4.76; *p* < 0.05), while no main effect of group or congruency by group interaction was found.

Participants were asked to fill out the Fagerström Test for Nicotine Dependence Questionnaire (Heatherton et al., 1991) after completing the experiment. The mean addiction score was 3.2; the severity of nicotine dependence was positively correlated with reaction times (*r* = 0.60; *p* = 0.02).

### ERP Data

To test for the NoGo N200 effect, ERPs following the presentation of the smoking-related pictures were analyzed, with non-smoker and smoker groups not separated. Testing the mean amplitude between 250 and 400 ms for F3, F4 and Fz electrodes with a 2 (Go/NoGo) × 2 (congruent/incongruent) × 3 (electrode: F3 vs. Fz vs. F4) repeated measures ANOVA revealed a significant Go/NoGo main effect (*F*_(1,29)_ = 28.90; *p* < 0.001; $\eta_{p}^{\text{2}}$ = 0.5), a significant electrode main effect (*F*_(2,58)_ = 4.92; *p* = 0.02; $\eta_{p}^{\text{2}}$ = 0.15), and a significant Go/NoGo × electrode interaction (*F*_(2,58)_ = 9.73; *p* < 0.001; $\eta_{p}^{\text{2}}$ = 0.25), while no main effect of congruency was found (*F*_(1,29)_ = 0.01; *p* = 0.9).

**Figure 3:**
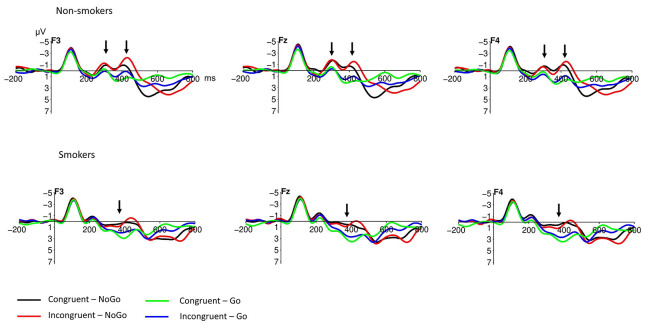
Stimulus-locked grand average event-related potential (ERP) waveforms at frontal electrode sites after the presentation of smoking-related pictures; for non-smokers and smokers. Note that the NoGo N200 seems to have two peaks, the second being delayed in the incongruent compared to the congruent condition—but only in the non-smoker, not in the smoker group (*pointing arrows*). The baseline used is −100 to 0 ms. The displayed waveforms were filtered with a 12 Hz low-pass filter.

Inspection of the ERPs to smoking-related pictures at frontal electrode positions separately for non-smokers and smokers (Figure 3) revealed that non-smokers show a delay of the N200 component in the incongruent condition, whereas smokers show no difference between the congruent and the incongruent condition.

**Figure 4:**
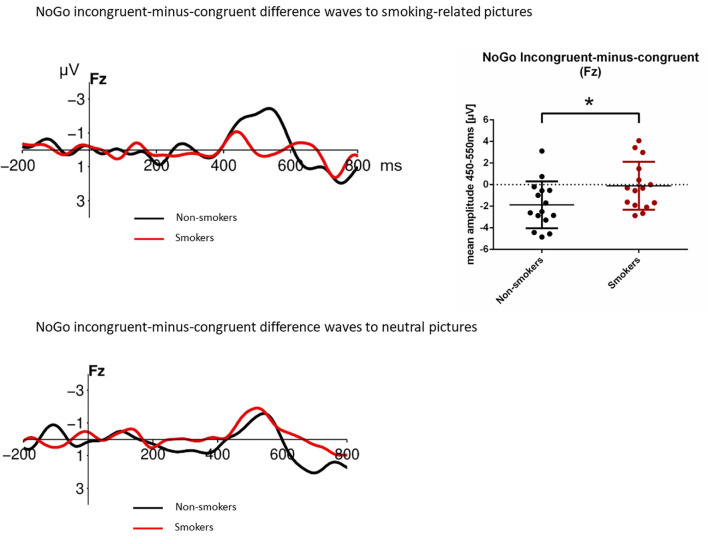
NoGo incongruent-minus-congruent difference waveforms in non-smokers and smokers to smoking-related pictures (top) and neutral pictures (down) at Fz. Note that the mean amplitude at 450–550 ms differs significantly between groups when smoking-related pictures are presented, while no difference between groups is observable after the presentation of neutral pictures. The displayed waveforms were filtered with a 12 Hz low-pass filter. * *p* < 0.05.

To test for this NoGo N200 difference between non-smokers and smokers, NoGo incongruent-minus-congruent difference waves to smoking-related pictures were calculated (Figure 4). Testing the mean amplitude between 450 and 550 ms for F3, F4 and Fz electrode with a 2 (group: non-smoker vs. smoker) × 3 (electrode: F3 vs. Fz vs. F4) repeated measures ANOVA revealed a marginally significant main effect of group (*F*_(1,14)_ = 3.84; *p* = 0.07; $\eta_{p}^{\text{2}}$ = 0.22) and a significant main effect of electrode (*F*_(2,28)_ = 7.78; *p* = 0.003; $\eta_{p}^{\text{2}}$ = 0.36), whereas the group × electrode interaction was not significant (*F*_(2,28)_ = 0.04; *p* = 0.96). Unpaired *t*-tests of the NoGo incongruent-minus-congruent mean amplitude between 450 and 550 ms for non-smokers vs. smokers revealed significant differences at F3 (*p* = 0.024), Fz (*p* = 0.037) and F4 electrode positions (*p* = 0.035). NoGo incongruent-minus-congruent difference waves to neutral/non-smoking pictures, in contrast, showed no significant difference between non-smokers and smokers.

## Discussion

We studied implicit attitudes towards smoking in smokers and non-smokers using a Go/NoGo association task (GNAT). Participants were shown either smoking-related or neutral (non-smoking-related) pictures requiring the same response as either positive or negative words according to systematically varied instructions.

Behaviorally, both smokers and non-smokers showed slower reaction times and higher error rates to smoking-related pictures in the incongruent condition (“press if a smoking-related picture or a positive word”/“press if a neutral picture or a negative word”), suggesting that both groups hold a negative automatic attitude towards smoking. This is in line with previous studies reporting similar (negative) implicit attitudes towards smoking in smokers and non-smokers alike (Swanson et al., 2001; Huijding et al., 2005; Glock et al., 2014; Cui et al., 2019), although findings vary depending on the social context and the duration of the cigarette deprivation, i.e., on craving (Wiers and De Jong, 2006; Payne et al., 2007; Waters et al., 2007; Woud et al., 2016). Importantly, implicit attitudes towards smoking seem to predict future smoking cessation (Chassin et al., 2010; Lee et al., 2017) and relapse (Spruyt et al., 2015).

Electrophysiologically, we found a robust N200 component in the NoGo condition after the presentation of smoking-related pictures in all participants. The NoGo N200 has consistently been described in Go-/NoGo (Jodo and Kayama, 1992) and GNAT experiments (Banfield et al., 2006; Wu et al., 2014) and is assumed to reflect response conflict monitoring (between the response tendency and the required withholding of response) by the ACC (Nieuwenhuis et al., 2003). It has been argued that conflict-related effects primarily impact the N200, whereas inhibition-related processes affect the P300 component (Enriquez-Geppert et al., 2010). In the context of the present study, we interpret the N200 component as a marker for conflict detection.

The NoGo N200 is delayed in trials with an incongruent pairing of stimuli compared to congruent pairings (Banfield et al., 2006; van der Lugt et al., 2012; Wu et al., 2014), which has been interpreted as the first manifestation of implicit attitude activation, taking place at 250–300 ms post-stimulus, whereas the behavioral response (reaction times, error rates) indicative of implicit associations becomes available considerably later, at about 600 ms.

Importantly, the present study identifies this delay of the NoGo N200 component in incongruent trials, but only in the non-smokers: whereas the NoGo N200 in the non-smoker group seems to have two peaks, with the second being pronounced and significantly delayed, within the smoker group there is no clear difference between the congruent and the incongruent conditions. This suggests that the implicit attitude activation (i.e., of a negative implicit attitude towards smoking), as manifested in the dissociation between the congruent and incongruent condition, is weaker or absent in the smoker group.

This is in partial contrast to the behavioral data which suggest that smokers and non-smokers hold negative implicit attitudes towards smoking, as reflected in prolonged reaction times and higher error rates in incongruent trials.

However, the observed behavioral and electrophysiological findings likely reflect different stages of (implicit) attitude formation and processing. The behavioral effect appears relatively late, at about 600 ms. At that time, negative implicit attitudes towards smoking are present in smokers as well as non-smokers. The NoGo N200, however, begins earlier, at about 250 ms. At that time, smokers, unlike non-smokers, may have not yet formed their negative attitude but instead, hold a more positive or ambivalent attitude towards smoking. Although conclusions are speculative, and further studies on the process of implicit attitude formation are needed, the data obtained in our study seem to corroborate the hypothesis that automatic or implicit attitudes are subject to changes or modulation within the first hundreds of milliseconds of their formation, and might be—probably in a predominantly involuntary manner—adjusted in the course of this process towards more socially acceptable attitudes (i.e., that smoking is bad).

This time course of implicit attitude formation is consistent with several previous studies. van der Lugt et al. (2012) found evidence of automatic attitude activation towards young and old people at 230 ms post-stimulus onset. Wu et al. (2014, 2016) describe implicit self-positivity to be activated and available in less than 300 ms. Osinsky et al. (2014) show that faces in social interaction are evaluated at 320 ms following the onset of face presentation.

It has to be noted that, when studying behavior or electrophysiology in addiction, the craving status should be considered. In our study, smokers were not allowed to smoke within 2 h before the start of our experiment, and one thus could expect that we induced (mild) craving. Additionally, our group was somewhat heterogeneous, as smokers who smoked seven up to 25 cigarettes per day were included, which could be associated with a different craving status. Participants were asked to fill out the Fagerström Test for Nicotine Dependence Questionnaire (Heatherton et al., 1991) after completing the experiment (showing a positive correlation with reaction times for smokers); however, the level of craving was not systematically assessed. Individuals craving to smoke have been reported to show impaired cognitive control (Donohue et al., 2020), and the current withdrawal state has been shown to influence smokers’ implicit attitudes towards smoking (less negative when the craving is higher; Payne et al., 2007). However, the fact that our behavioral data suggest that both non-smokers and smokers hold equally negative implicit attitudes towards smoking speaks for only a mild withdrawal status of our participating smokers.

In summary, the data obtained in our study is interpreted as providing evidence that smokers hold a positive or at least ambivalent implicit attitude towards smoking—in the sense of a rather pre-reflexive evaluative disposition—, which is activated at 300–400 ms post-stimulus presentation. However, this automatic or implicit attitude seems to change over (short) time—presumably due to social expectations—, so that at 600–700 ms, smokers show behavioral evidence for a negative implicit attitude towards smoking, similar to non-smokers. These findings are in line with a study from De Houwer et al. (2006), which reported that smokers show positive implicit attitudes using a personalized version of the IAT that is purportedly less sensitive to socio-cultural influences than the standard IAT. They also observed a similar positive effect for smokers using an approach-avoid response, which suggested that smoking is related more to wanting than to liking (see Palfai and Ostafin, 2003). To conclude, positive implicit attitudes—as indicated by the lack of delay of the NoGo N200 component in incongruent trials in the smoker group—could play a considerable causal role in the maintenance of smoking behavior after all.

## Data Availability Statement

The raw data supporting the conclusions of this article will be made available by the authors, without undue reservation.

## Ethics Statement

The studies involving human participants were reviewed and approved by Ethics Committee of the Otto von Guericke University Magdeburg, Magdeburg. The patients/participants provided their written informed consent to participate in this study.

## Author Contributions

TW-A, AL, JB, JD, MH, and TM: design/conductance of the study and data analysis. TW-A: first draft of the manuscript. AL, JB, JD, AC, MH, and TM: critical revision of the manuscript. All authors contributed to the article and approved the submitted version.

## Conflict of Interest

The authors declare that the research was conducted in the absence of any commercial or financial relationships that could be construed as a potential conflict of interest.

## Footnotes

^1^https://www.jamovi.org
