# Supplementary material for: Studying Implicit Attitudes Towards Smoking: Event-Related Potentials in the Go/NoGo Association Task
Source: Front Hum Neurosci. 2021 Feb 5;15:634994. doi: 10.3389/fnhum.2021.634994 (PMC7892465; doi:10.3389/fnhum.2021.634994)
Supplement: Supplementary file 1 [file Table_1.DOCX]

**Appendix**

**A. List of word stimuli used in the GNAT (originally in German)**

Positive words:

adventurous, appetizing, applause, excitement, excellent, talented, happy, helpful, popular, reward, flowers, fabulous, fantastic, holiday, joy, cheerfulness, hospitable, enjoyable, healthy, happiness excellent, heart-warming, humor, jackpot, kiss, laughing, life-hungry, delicious, lovingly, sweet, paradise, silk, sun, sunrise, sunshine, top, super, sympathetic, dancing, triumph, embrace, confidence, wonderful.

[abenteuerlustig, appetitlich, Applaus, Aufgeregtheit, ausgezeichnet, begabt, Beglückung, behilflich, beliebt, Belohnung, Blumen, fabelhaft, fantastisch, Feiertag, Freude, Fröhlichkeit, gastfreundlich, genüsslich, gesund, Glück, hervorragend, herzerfreuend, Humor, Jackpot, Kuss, lachend, lebenshungrig, lecker, liebevoll, niedlich, Paradies, Seide, Sonne, Sonnenaufgang, Sonnenschein, spitze, super, sympathisch, Tanzen, Triumph, Umarmung, Vertrauen, wunderbar.]

Negative words:

aversion, loathsome, aggressive, fearful, scary, offensive, annoying, arrogant, extermination, threat, funeral, bombs, evil, malicious, brutal, disaster, self-centered, selfish, conceited, vain, disgusting, torture, dangerous, prison, hatred, cruel, hate, heartless, snobbish, hell, disease, miserable, murder, slaughterhouse, dirty, death, agony, manslaughter, tragic, sad, crime, disgusting, toothache, destruction, angry.

[Abneigung, abscheulich, aggressiv, ängstlich, anstößig, ärgerlich, arrogant, Ausrottung, Bedrohung, Beerdigung, Bomben, böse, boshaft, brutal, Desaster, egozentrisch, eigennützig, eingebildet, eitel, ekelig, Folter, gefährlich, Gefängnis, Gehässigkeit, grausam, Hass, herzlos, hochnäsig, Hölle, Krankheit, miserable, Mord, Schlachthaus, schmutzig, Tod, Todeskampf, Totschlag, tragisch, traurig, Verbrechen, widerlich, Zahnlöcher, Zerstörung, zornig.]

**B. Examples for smoking pictures (left column) and neutral pictures (right column)**

See supplemental image file.
