# Supplementary figures and images for: Studying Implicit Attitudes Towards Smoking: Event-Related Potentials in the Go/NoGo Association Task
Source: Front Hum Neurosci. 2021 Feb 5;15:634994. doi: 10.3389/fnhum.2021.634994 (PMC7892465; doi:10.3389/fnhum.2021.634994)

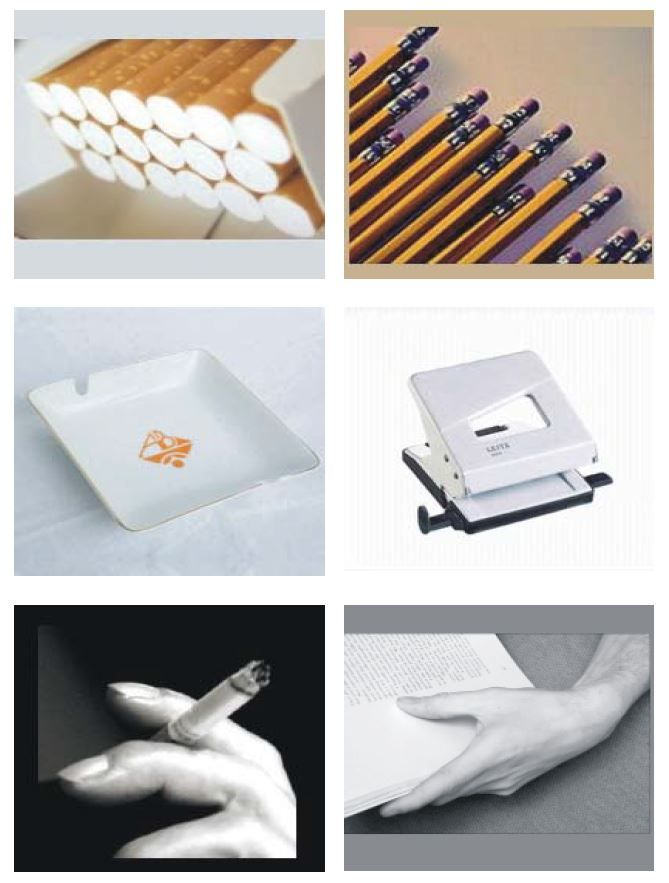

Supplement: Supplementary file 2 [file Image_1.TIF]
